# High-Density Electromyography Provides Improved Understanding of Muscle Function for Those With Amputation

**DOI:** 10.3389/fmedt.2021.690285

**Published:** 2021-08-09

**Authors:** Usha Kuruganti, Ashirbad Pradhan, Jacqueline Toner

**Affiliations:** ^1^Andrew and Marjorie McCain Human Performance Laboratory, Faculty of Kinesiology, University of New Brunswick, Fredericton, NB, Canada; ^2^Waterloo Engineering Bionics Lab, University of Waterloo, Waterloo, ON, Canada; ^3^Faculty of Health, Dalhousie University, Halifax, NS, Canada

**Keywords:** high-density electromyography, biological signal processing, prosthetics, surface electromyography, neuromuscular function, spatial muscle activity

## Abstract

Transtibial amputation can significantly impact an individual's quality of life including the completion of activities of daily living. Those with lower limb amputations can harness the electrical activity from their amputated limb muscles for myoelectric control of a powered prosthesis. While these devices use residual muscles from transtibial-amputated limb as an input to the controller, there is little research characterizing the changes in surface electromyography (sEMG) signal generated by the upper leg muscles. Traditional surface EMG is limited in the number of electrode sites while high-density surface EMG (HDsEMG) uses multiple electrode sites to gather more information from the muscle. This technique is promising for not only the development of myoelectric-controlled prostheses but also advancing our knowledge of muscle behavior with clinical populations, including post-amputation. The HDsEMG signal can be used to develop spatial activation maps and features of these maps can be used to gain valuable insight into muscle behavior. Spatial features of HDsEMG can provide information regarding muscle activation, muscle fiber heterogeneity, and changes in muscle distribution and can be used to estimate properties of both the amputated limb and intact limb. While there are a few studies that have examined HDsEMG in amputated lower limbs they have been limited to movements such as gait. The purpose of this study was to examine the quadriceps muscle during a slow, moderate and fast isokinetic knee extensions from a control group as well as a clinical patient with a transtibial amputation. HDsEMG was collected from the quadriceps of the dominant leg of 14 young, healthy males (mean age = 25.5 ± 7 years old). Signals were collected from both the intact and amputated limb muscle of a 23 year old clinical participant to examine differences between the affected and unaffected leg. It was found that there were differences between the intact and amputated limb limb of the clinical participant with respect to muscle activation and muscle heterogeneity. While this study was limited to one clinical participant, it is important to note the differences in muscle behavior between the intact and amputated limb limb. Understanding these differences will help to improve training protocols for those with amputation.

## Introduction

Transtibial amputation can negatively impact an individual including effects to their body image, vocation and ability to socialize (1). Associated changes in the individual's anatomical structure can also lead to asymmetries in the musculature of the lower limbs leading to alterations in individual's gait and reductions in activity levels. The risk of developing secondary complications such as osteoarthritis after a transtibial amputation suggests there should be more focus on performance assessments of the lower limb musculature during rehabilitation. These assessments can be measured safely with the use of an isokinetic dynamometer and can produce accurate muscle strength results for both dynamic and static conditions (2). An isokinetic muscle contraction occurs when the muscle shortens and contracts as it goes through a range of motion at a constant speed. Using an isokinetic dynamometer, participants can complete an isokinetic exercise throughout their full range of motion at a set angular velocity (3). Isokinetic dynamometry has become a useful tool in research studies, rehabilitation, and training settings. It allows clinicians to obtain quantitative data about the dynamic contraction of muscles with a controlled speed, demonstrating the individual's areas of strength improvement or strength discrepancies. Currently, the isokinetic dynamometer is considered to be the gold standard as it is safe and can be used in a controlled environment and has been used successfully with prosthesis-wearing individuals with transtibial amputation to examine the knee extensors and flexors (4, 5).

While surface EMG captures gross muscle activity rather than individual motor units (MUs), it can still be used to evaluate muscle properties including muscle affected by disuse or disorder. There are numerous signal processing techniques that have been used to estimate the amplitude of EMG signal including averaging, smoothing, using the integrated signal, as well as rectification and calculation of the root mean square (RMS) value. Traditional sEMG has been limited as it reflects the activity of the muscle directly below the recording electrode. Linear array and two-dimensional multichannel EMG systems have been promising for a detailed investigation (e.g., spatial information) of muscle behavior. While linear arrays involve connecting several bipolar electrodes along a line, 2D multichannel EMG systems increase the number of electrodes and their recording positions (6). These types of recordings are particularly well-suited for clinical applications including those individuals with amputated limbs. Multichannel EMG systems can better represent muscle activation and estimated force compared to single-channel systems (7) and can also provide complementary spatial information (8–10). This spatial data can be used to create two-dimensional topographical color maps, or “activation maps,” that can then be used to examine muscle activity patterns during different tasks (11, 12). Multichannel EMG has also been shown to be effective to non-invasively investigate MU activation of muscle during force production at varying levels of force contraction (13). These multichannel recordings, often termed “high-density” surface EMG (HDsEMG) have been used to examine motor unit recruitment, recruitment patterns and changes in recruitment (13–18). The HDsEMG maps represent the spatial distribution of intensities of active MUs over the surface of the muscle (19).

Surface EMG in individuals with amputations is limited by the difficulty in electrode placement as there are often changes in the muscle origin due to atrophy or complications from surgery. While HDsEMG has been shown to be reliable and provide significant data to better understand muscle activation, its use with those with amputated limbs remains limited and to a greater extent the technology has been used in upper limbs (9, 10, 20). In those studies the HDsEMG signal was used primarily for pattern classification of a prosthesis. Since then, there are have been advances in the features used to examine HDsEMG maps and it has been shown that several spatial parameters can provide helpful information regarding muscle activation including amplitude, frequency, motor unit conduction velocity, motor unit size, and signal entropy (14, 21). Other spatial features that can be used to characterize muscle activation include signal entropy (13, 14, 22), intensity, and the center of gravity or centroid (19, 23).

The use of HDsEMG has increased rapidly over the last decade although surprisingly, there are few studies that have used surface EMG to examine muscle activation patterns in those with amputated lower limbs and, in particular, both the intact limb and residual limb (24, 25). Although there has been promising work using EMG from the ampuated upper limb for myoelectric control, few studies have used this technology to characterize lower limb muscle activity (26). A previous study using HDsEMG for lower limb monitored the muscle activity during isometric contraction (26). Another study used bipolar electrodes to assess the EMG activity of the thigh as well as the residual leg segment of the lower limb during gait (24). To our knowledge, there are no studies that have investigated quadriceps activity of the transtibial-ampuated leg muscle activity using HDsEMG during other dynamic activities such as isokinetic knee extension which could help clinicians when developing rehabilitation programs for this population.

Employing HDsEMG during isokinetic movements can be used to understand and quantify changes in the spatial distribution of muscle activity with and without amputation. This can help highlight muscle differences due to amputation and also help develop improved rehabilitation protocols. It has been shown that several spatial parameters can provide helpful information regarding muscle activation including amplitude, frequency, motor unit conduction velocity, motor unit size, and signal entropy (14, 21). Features such as entropy and coefficient of variation (CV) have been used to characterize muscle fiber heterogeneity in spatial EMG distribution (18, 27). The change in the distribution of muscle activity during either sustained or dynamic contractions can be evaluated by quanitfiying a shift in the centroid of the HDsEMG amplitude map, the point which defines the barycentre of muscle activation (14, 19, 23, 28, 29). In addition, it has been shown that regional changes can occur in muscle activation at different intensities (30). Examining the CoG across different contraction speeds will help to determine the effect of speed on these regional muscle activation changes and if it differs due to amputated musculature.

The purpose of the present work was to examine spatial features of the quadriceps HDsEMG during isokinetic knee extensions across a range of speeds for 14 able-bodied males as well as one male clinical participant. The spatial features were also compared between intact and ampuated limb after transtibial amputation to highlight muscle differences. HDsEMG spatial parameters were also compared between the clinical participant and an age-matched control participant. In this work we also present an additional feature of the centroid using a trajectory-based analysis using the entire contraction, thereby examining the x and y variations of the centroid across the movement. Previously the center of gravity (CoG) of the spatial activation map has been estimated as the x- and y-coordinates at the peak intensity. This has been limited to one time-point and it is possible that some information is lost by this reduction. The trajectory-based analysis records the CoG of the entire contraction duration suitable for investigating dynamic movements.

The prevalence of lower limb amputations has increased steadily (31) and there is a need to better understand the neuromuscular differences between healthy and amputated leg muscle function. Comparison of spatial HDsEMG features between the intact and amputated limb will provide significant information regarding differences due to amputation and will help to develop more targeted rehabilitation programs. Isokinetic dynamometery is routinely used in clinical settings and the data obtained in this work will help to provide insight on muscle behavior that will help to develop strength training and testing protocols for those with lower limb amputations.

## Materials and Methods

### Participants

Fourteen healthy, able-bodied males (mean age = 25.5 ± 7 years old, mean height = 1.8 ± 0.1 m, mean weight = 81.8 ± 11.0 kg) were recruited for this study. Table 1 provides the details regarding the leg measurements (skinfold and upper thigh circumference). All participants identified their right sides as dominant and further anthropometrics for the able-bodied group are presented in Table 1.

**Table 1:** Skinfold measurements (mm) and upper thigh circumference (cm) of the right leg for the control group.

**Anterior thigh (mm)**	**Posterior thigh (mm)**	**Patella (mm)**	**Upper thigh circumference (cm)**
15.2 ± 9	15.4 ± 10	13.8 ± 11	52.7 ± 5

*Values are Mean ± SD*.

One clinical participant with a transtibial amputation was recruited for this study. The clinical participant was a 23-year-old male (height = 1.8 m, weight = 75.5 kg) with a right transtibial amputation resulting from trauma. He had been using his lower limb prosthesis for ~7.5 years prior to the study testing. He identified his intact side (left) as his dominant side. The clinical participant was able to ambulate independently without the use of an assistive device (e.g., crutch or cane). Clinical participant anthropometrics are presented in Table 2.

**Table 2:** Skinfold measurements (mm) and upper thigh circumference (cm) for the clinical participant.

**Side**	**Anterior thigh (mm)**	**Posterior thigh (mm)**	**Patella (mm)**	**Upper thigh circumference (cm)**
Affected side (right)	16.0	14.0	14.0	43.5
Sound side (left)	21.0	22.0	19.0	50.0

*The affected (amputated) side was the right side and considered the participant's non-dominant side and the sound side was the left side (dominant) side*.

### Experimental Design

Prior to testing, participants were assessed using the following criteria: (1) ambulatory without a mobility aid; (2) absence of injury in the last 6 months; (3) absence of health complications that would create a risk of injury during the study; and (4) absence of inflammation and pain of the knee joint. All participants were given an overview of the data collection equipment, procedures, and information on any risks associated with testing. Participants were asked to read and sign an informed consent form provided by the experimenter. The clinical participant was also asked for information regarding his amputation and prosthesis including the date of amputation, reason for amputation, activity level, chronic conditions, medical history, and the “Get Active” questionnaire. Preliminary measurements were taken, and the prosthesis was examined. This research study was approved by the University of New Brunswick's Research Ethics Board.

### Experimental Protocol

Participants were asked to walk around a track for 5 min at a comfortable speed to warm up muscles prior to any testing. All testing were conducted in Human Performance Laboratory. Once the warm-up was completed, the participant was asked to be seated in the chair of an isokinetic dynamometer (Cybex, Humac Norm). A 64-channel semi-disposable electrode grid (ELSCH064NM2, OT Bioelecttronica, Italy 13 rows × 5 columns) was placed over the rectus femoris of the participant's dominant leg. A double-sided adhesive foam grid was set on the electrode grid, a conductive cream was spread and into each cavity to create skin contact with each electrode. The rectus femoris of the participant's dominant leg was palpated and the electrode grid was placed over the muscle. For the clinical participant, a grid was placed over both their unaffected limb and the amputated side for comparison. A pre-gelled AgAgCl ground electrode (Duo-Trode, Myotronics Inc., WA, USA, diameter = 12.5 mm) was placed over the patella for all participants. Prior to electrode placement, the area was shaved and cleaned. Multichannel High-Density Surface Electromyography (HDsEMG) data were recorded during all contractions at 1,024 Hz using a wireless high-density EMG (Sessantaquattro, OT Bioelettronica, Italy, input impedance: >90 MΩ, CMMR: >96 dB, filter: 10 Hz low cut-off, 500 Hz high cut-off, noise: <2 μV_RMS_).

**Figure 1:**
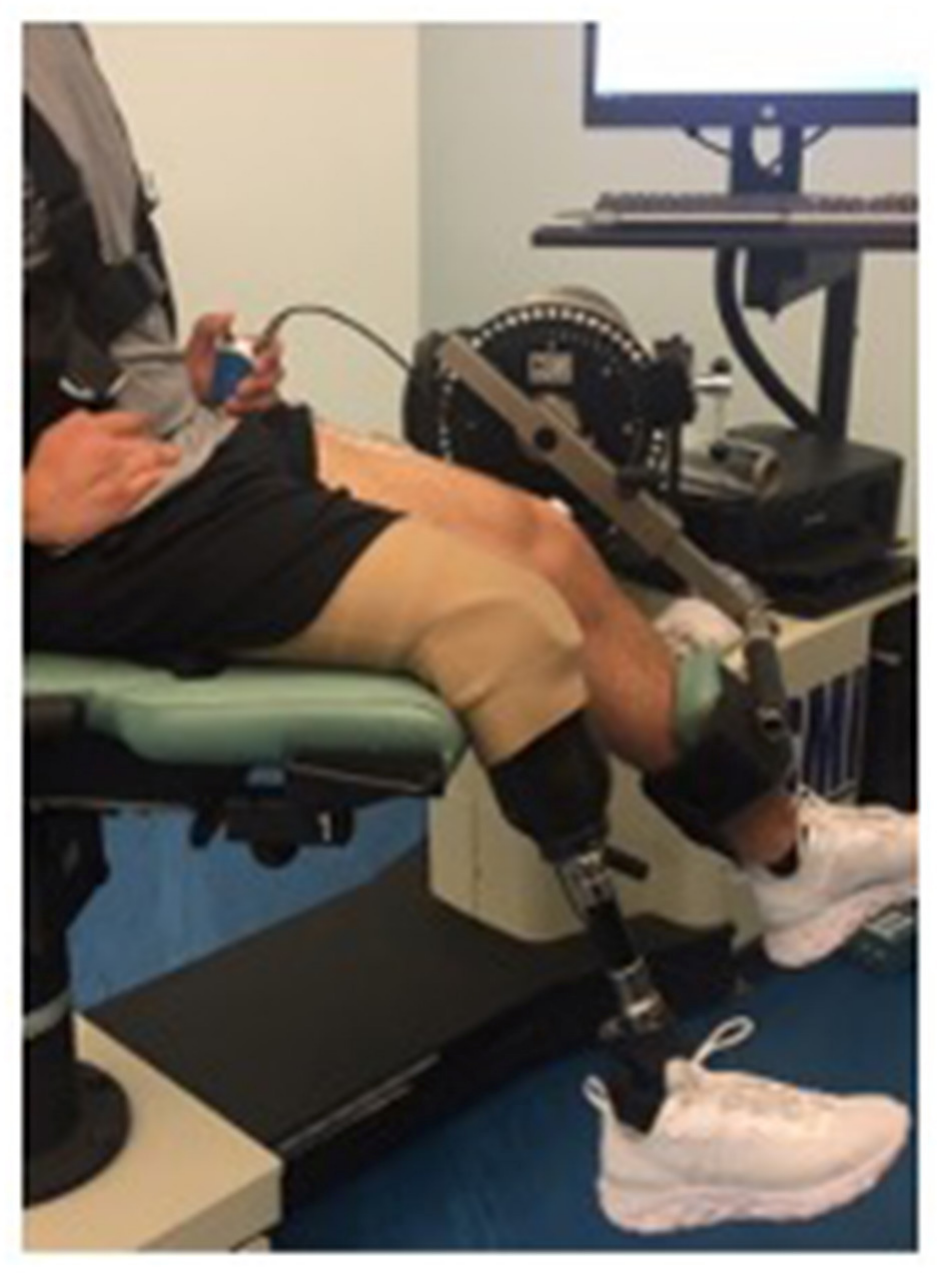
Clinical participant during testing of isokinetic knee extension.

The participant was situated in the dynamometer in a comfortable seating position (Figure 1). The fulcrum of the dynamometer was aligned to the rotational axis of the knee using the lateral femoral condyle (32). The participant was secured to the chair of the dynamometer using straps around their chest. The knee extension adapter was secured ~2 inches above the ankle joint. The participant was then asked to perform an isometric maximum voluntary contraction (MVC) for 5-s with the dynamometer set to a speed of 0°/s and a knee angle of ~90°. The clinical participant completed trials of their affected side while wearing their prosthesis. The knee extension adapter was secured to their artificial leg ~3 inches above the top of their shoe. The clincal participant was then asked to complete an MVC similar to the control participants. The clinical participant was also asked to complete the contractions with their unaffected limb to compare with their ampuated side.

To ensure that the clinical participant was comfortable in the dynamometer and that the torque data was reliable, he was asked to visit the lab twice to complete the assessment. The two visits were separated by 2 weeks.

Participants were then provided an opportunity to practice isokinetic submaximal contractions. They were then asked to produce isokinetic contractions, randomly, at three speeds: slow (60°/s), moderate (90°/s), and fast (120°/s). Participants were asked to complete two trials at each speed with 10-min rests between each contraction. All torque data were recorded at a frequency of 100 Hz.

### Data Analysis

HDsEMG data was filtered with a bandpass filter 20–400 Hz and the Root Mean Square (RMS) was calculated for every 1-s interval for each channel (OT Biolab). EMG data were analyzed using a 250 ms epoch centered at the midpoint of the contraction. The signals were anlayzed off-line using custom-built open-source MATLAB software (41).

**Figure 2:**
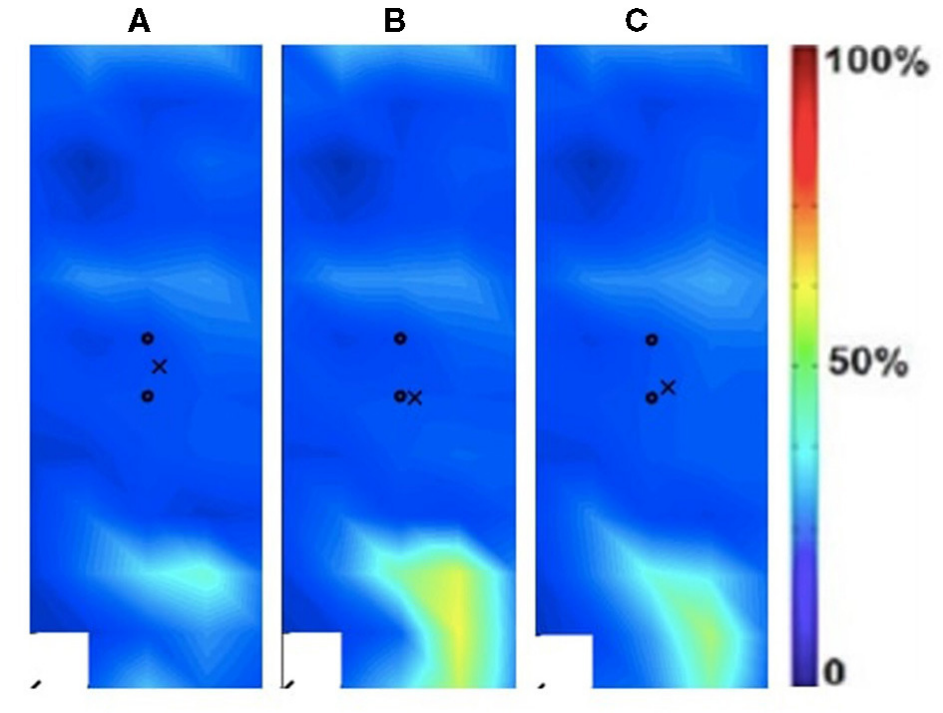
Activation map of clinical participant (male) of the affected (right) rectus femoris during the following three velocities. **(A)** 60°/s; **(B)** 90°/s; **(C)** 120°/s.

Spatial distribution was estimated using the Root Mean Square (RMS) EMG signals for each of the electrode grid locations from which 2D maps were developed (Figure 2). The 2D maps represent the intensity of muscle activation over the surface of the muscle (19):


(1) $$\begin{array}{l} HM_{ij}=RMS\;\left(sEMG_{ij}\right) \end{array}$$

where HM is an activation map and each pixel in a map (HM_ij_) corresponds to an RMS value of a channel in an electrode array (position i,j). Five time features and one frequency feature were calculated from the activation map for analysis: intensity, differential intensity, modified entropy, coefficient of variation (COV), center of gravity (CoG) and median frequency.

It has been suggested previously that the relationship between EMG amplitude and force generated is not linear (33, 34) and therefore intensity was defined similar to previous work (19, 35) as the common logarithm of the mean intensity of the HDsEMG maps:


(2) $$\begin{array}{l} I={\text{log}}_{10}\frac{1}{N}\underset{i,j}{\sum }\text{H}{\text{M}}_{\text{i},\text{j}} \end{array}$$

where I is an intensity feature calculated from the HDsEMG activation map HM with a total number of N channels, and HM_i,j_ is the intensity of a channel located at position i,j.

Intensity of a single differential channel or differential intensity (DI) was calculated as a common logarithm of an RMS value of difference of two consecutive channels in the direction of the muscle fibers (19):


(3) $$\begin{array}{l} DI=log_{10}\left(RMS\left(sEMG_{i,j}-sEMG_{i+1,j}\right)\right) \end{array}$$

Entropy and coefficient of variation (CoV) were used to represent the increased variety of signals (heterogeneity) of the HDsEMG distribution. When all of the channels show the same RMS values, maximum entropy occurs. When all of the channels except for one have an RMS value of zero, minimum entropy occurs. Therefore, as entropy increases, heterogeneity of the muscle fiber types decreases.


(4) $$\begin{array}{l} \text{E}=-\overset{59}{\underset{i=1}{\sum }}{p\left(i\right)}^{2}\;log_{2}\;{p\left(i\right)}^{2} \end{array}$$

Where the square of the RMS value at electrode i, p*(i)*, is then normalized by the sum of the squares of all 59 RMS values (14).

CoV was calculated by taking the standard deviation (SD) of the 59 RMS values divided by the average of the 59 RMS values. The CoV value is small when the SD is small relative to the mean which occurs when the channels are more uniform. A smaller CoV value implies low heterogeneity and therefore homogeneity of the muscle fibers. All the signal processing and evaluation was performed using a custom-designed MATLAB software (18).

The centroid of the activation map or the center of gravity (CoG) has been used to describe the localization of the muscle activity. The centroid coordinates of the maps describe muscle adaptation, defined as CoG_x_ and CoG_y_ for the medial-lateral and cranial-caudal direction of the map (29).

The center of gravity (CoG) of an HDsEMG map was calculated as (23):


(5) $$\begin{array}{l} CoG\left[\begin{array}{c} x\\ y \end{array}\right]=\frac{1}{\sum_{i,j}HM_{i,j}}\underset{i,j}{\sum }HM_{i,j}\left[\begin{array}{c} i\\ j \end{array}\right] \end{array}$$

where CoG_x_ and CoG_y_ represent the x and y coordinates of HM. The previous studies utilize a mid-point-based analysis for isometric contractions (19) or a peak-force based analysis based on the spatial activity map for isokinetic contractions. The CoG_x_ and CoG_y_ values were reported for the contraction trials.

The present study also suggested a CoG-trajectory based analysis that recorded the CoG values from the beginning to the end of the contraction. The CoG-trajectory was calculated as:


(6) $$\begin{array}{l} CoG_{traj}\left(t\right)\left[\begin{array}{c} x\\ y \end{array}\right]\\ \;=\frac{1}{\sum_{i,j}{HM\left(t\right)}_{i,j}}\underset{i,j}{\sum }{HM\left(t\right)}_{i,j}{\left[\begin{array}{c} i\\ j \end{array}\right]}_{t=contraction\;duration} \end{array}$$

The CoG_traj_ provides additional information such as the variation of the x and y coordinates over the entire contraction. For further analysis, the mean, standard deviation, and min-max range were reported for each contraction trial.

The frequency feature, median frequency is the parameter that divides total power into two equal parts: and was calculated as:


(7) $$\begin{array}{l} \int_{0}^{f_{med}}P\left(f\right)df=\;\frac{1}{2}\int_{0}^{\frac{f_{s}}{2}}P\left(f\right)df \end{array}$$

### Statistical Analysis

Prior to statistical analysis, Shapiro-Wilks tests were completed to ensure the normal distribution of the data. Following the confirmation of a normal distribution, pairwise *t*-tests with the Bonferroni correction method were used to examine differences in features due to speed with an alpha level set to 0.05. Center of Gravity (CoG) data is presented separately to compare the two methods. A Pearson product-moment correlation analysis was conducted to compare the two methods. Data are presented as the mean and SD for the able-bodied group for all features, with notes on significance. For the single clinical participant, descriptive data only is provided for comparison. All of the statistical tests were performed using RStudio 1.0. 136 (RStudio, Boston, MA).

## Results

### Spatial Features Rather Than Entropy, CoV, Intensity Mean RMS, and Median Frequency

The spatial features of entropy, CoV, intensity, mean RMS as well as median frequency are provided for both the able-bodied group and the clinical participant. Table 3 provides the mean data for these spatial features for the dominant side. A paired *t*-test was used to examine differences due to speed (0, 60, 90, 120°/s) with an alpha level set to 0.05 and no statistically significant differences were detected due to speed. Table 4 provides the same set of features for the clinical participant but for both the intact and affected side.

**Table 3:** Mean spatial features for the able-bodied group.

**Speed (°/s)**	**Entropy**	**CoV**	**Intensity**	**Differential intensity**	**Mean RMS**	**Median frequency**
60	5.58 ± 0.1	33.97 ± 6.45	−1.32 ± 0.4	−1.62 ± 0.4	0.07± 0.7	82.78 ± 18
90	5.54 ± 0.2	36.02 ± 25.1	−1.50 ± 0.4	−1.74 ± 0.4	0.04 ± 0.3	84.98 ± 17
120	5.52 ± 0.2	36.50 ± 24.8	−1.53 ± 0.4	−1.79 ± 0.4	0.04 ± 0.3	84.07 ± 14

*Entropy (a.u.), CoV (a.u.), intensity, differential intensity, and the mean RMS values of the right rectus femoris muscle in the control group individuals during the isokinetic knee extensions at three speeds. Values are Mean ± SD*.

**Table 4:** Mean spatial features for the clinical participant.

**Muscle side**	**Speed**	**Entropy**	**CoV**	**Intensity**	**Differential intensity**	**Mean_RMS**	**Median_frequency**
Affected (right)	60	5.3178	48.036	−0.91962	−0.98722	0.12033	71.331
	90	5.3972	42.955	−0.96957	−0.98503	0.10726	80.197
	120	5.381	43.559	−0.99619	−0.94454	0.10088	76.571
Intact (left)	60	5.5572	37.052	−0.69797	−0.81649	0.20046	63.474
	90	5.2689	51.34	−0.46778	−0.65402	0.34058	65.397
	120	5.1853	53.279	−0.64043	−0.70794	0.22886	82.364

*Entropy (a.u.), CoV (a.u.), and the mean RMS values of the rectus femoris muscle in the prosthesis user for the affected and intact side*.

Table 5 presents the differences in torque and EMG data between the prosthesis user and age-matched control participant. The prosthesis user had lower torque in his affected side than the non-dominant leg of the control participant. For both the clinical and control participants, as speed increased, generally torque decreased. The age-matched control participant did show little change due to speed on their dominant leg. Mean RMS was generally greater for the clinical participant compared to the age-matched control as were intensity and differential intensity. Median frequency was lower in the slow and moderate contractions and higher during the fast contraction for the clinical participant.

**Table 5:** Spatial activity map features of clinical and age-matched control participant.

	**Affected limb vs. non-dominant**		**Sound side vs. dominant**	
	**Clinical**	**Age-matched control**	**Clinical**	**Age-matched control**
**Peak torque (Nm)**				
60°/s	100.3	154.3	179.1	164.2
90°/s	85.4	102	148.2	161.9
120°/s	73.1	82.5	120.5	161.7
**Entropy (a.u.)**				
60°/s	5.32	5.76	5.56	3.95
90°/s	5.40	5.77	5.27	3.65
120°/s	5.38	5.21	5.19	4.82
**CoV (a.u.)**				
60°/s	48.04	21.19	37.05	90.32
90°/s	42.96	20.57	51.34	97.3
120°/s	43.56	52.17	53.28	65.55
**Intensity**				
60°/s	−0.920	−1.247	−0.698	−1.012
90°/s	−0.970	−1.311	−0.468	−0.966
120°/s	−0.996	−1.001	−0.640	−0.957
**Differential intensity**				
60°/s	−0.987	−1.270	−0.816	−1.100
90°/s	−0.985	−1.431	−0.654	−1.093
120°/s	−0.945	−0.923	−0.708	−1.001
**Mean RMS (mV)**				
60°/s	0.120	0.057	0.200	0.097
90°/s	0.107	0.049	0.341	0.108
120°/s	0.101	0.010	0.229	0.110
**Median frequency (Hz)**				
60°/s	71.3	95.4	63.5	70.6
90°/s	80.2	87.8	65.4	75.3
120°/s	76.6	46.2	82.4	66.1

*The features are shown for both the affected (prosthesis) and sound side across three speeds*.

### Spatial Activation Maps

**Figure 3:**
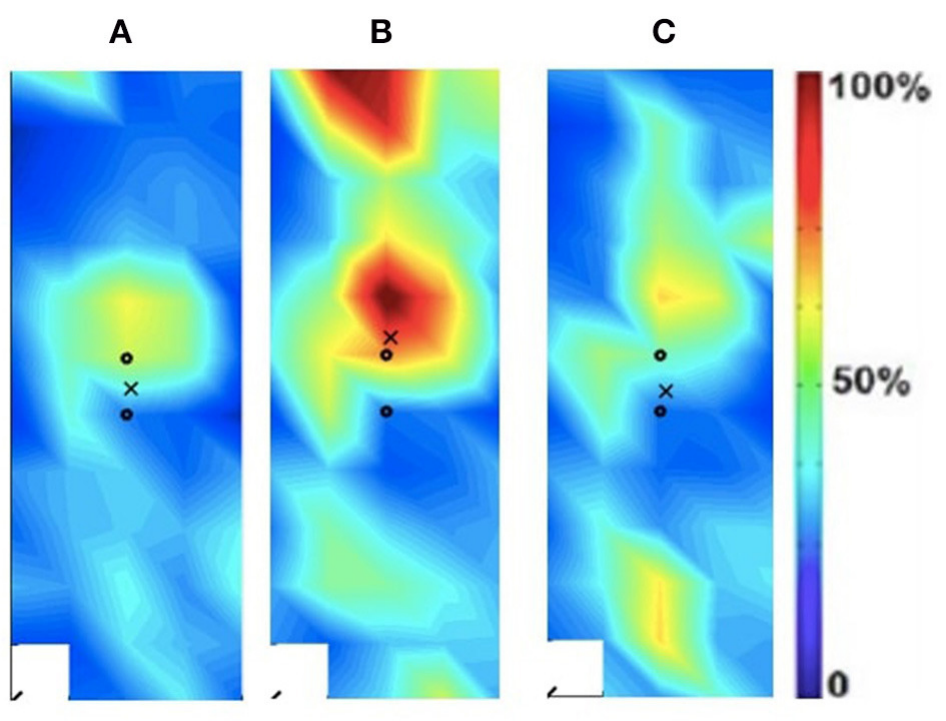
Activation map of prosthesis user participant (male) of the sound (left) rectus femoris during the following three velocities. **(A)** 60°/s; **(B)** 90°/s; **(C)** 120°/s.

The spatial maps for the clinical participant were compared between the intact and ampuated limb legs. Figures 2, 3 provide the activation maps generated for the clinical participant. Figure 2 shows the affected side while Figure 3 presents the sound limb at the three isokinetic speeds. The sound side of the clinical participant showed lower overall muscle activation compared to the amputated side. Figures 2, 3 illustrate the spatial activity of the clinical participant from the affected side (Figure 2) and sound side (Figure 3). These figures indicate that there is greater spatial activity in the sound side compared to the affected side. In addition, the pattern changes in the sound side due to speed with the moderate contraction indicating greater intensity (demonstrated by the areas of orange and dark red on the activity map). These changes are not observed on the affected side of the prosthesis user (Figure 2).

**Figure 4:**
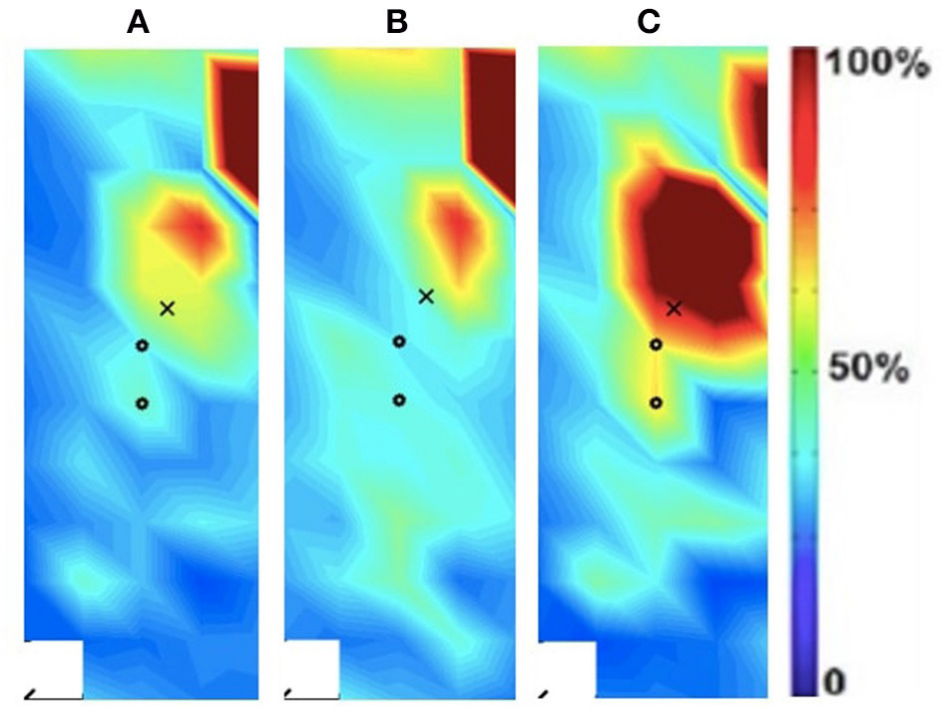
Activation map of age-matched control (male) of the dominant (right) rectus femoris during the following three velocities. **(A)** 60°/s; **(B)** 90°/s; **(C)** 120°/s.

**Figure 5:**
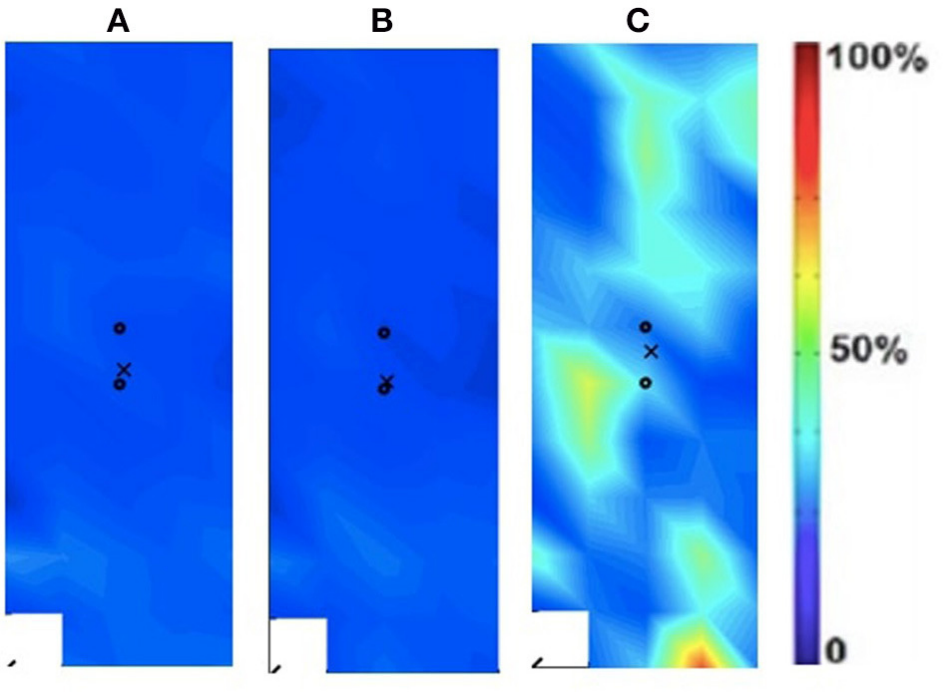
Activation map of age-matched control (male) of the non-dominant (left) rectus femoris during the following three velocities. **(A)** 60°/s; **(B)** 90°/s; **(C)** 120°/s.

In addition to the changes between the intact and ampuated limb, the prosthesis user was compared to an age-matched control participant. Figures 4, 5 provide the activation maps of the age-matched control participant. For the sound side, the clinical participant (Figure 3) was compared to the dominant side of the control (Figure 4) participant. Similar intensity patterns can be observed across all three speeds with the control exhibiting greater intensity (larger region of red) in the fast contraction (120°/s). For the affected side, the clinical participant (Figure 2) was compared to the non-dominant side of the control participant (Figure 5). It was observed that the clinical participant had similar patterns, with a few areas of greater activation (as indicated by the lighter blue color) at the 60 and 90°/s contractions, however a the higher speed of 120, the control participant had more activation across the RF.

The spatial features corresponding with the maps are provided in Table 5. The clinical participant's affected (prosthesis) side was compared to the non-dominant side of the age-matched control participant while their sound side (i.e., intact limb) was compared to the dominant side of the age-matched participant. The clinical participant exhibited lower peak torque and higher entropy, CoV, intensity, differential intensity and mean RMS compared to the age-matched control participant on their affected side. On their sound side the clinical participant demonstrated higher peak torque, entropy, differential intensity, intensity and mean RMS, and a lower CoV.

### Center of Gravity

**Figure 6:**
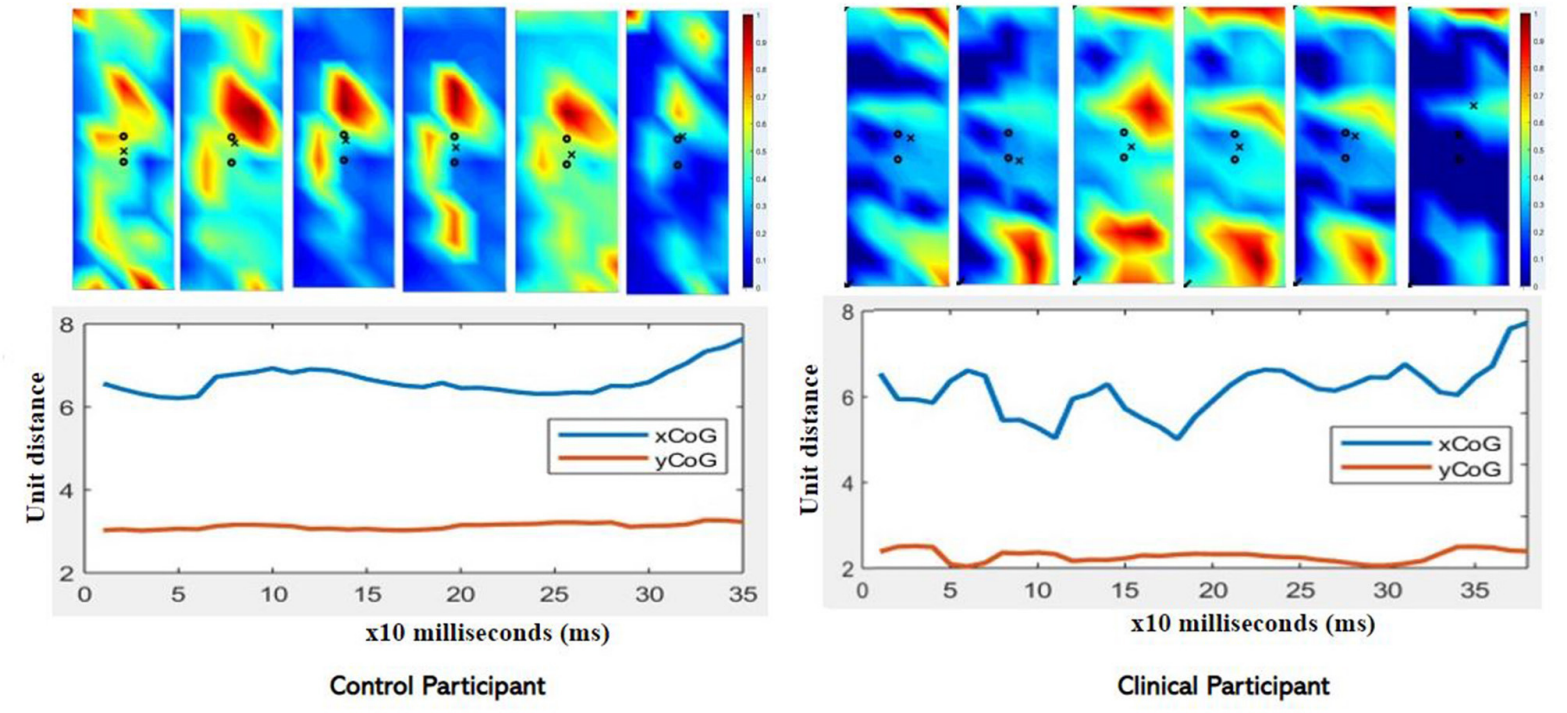
Comparison of CoGx_mean_ and CoGy_mean_ for control and clinical participant during moderate (90/s) isokinetic knee extension. Note the greater variability in the clinical participant compared to the control (age-matched) participant. Clinical participant also demonstrates reduced intensity as exhibited in the color map.

The CoG and CoG were calculated for each speed condition and each method, single point at peak intensity (CoG_x_ and CoG_y_) and using the trajectory (CoG_xmean_ and CoG_ymean_). For the trajectory method variance and range are provided in addition to the mean data. Table 6 presents the CoG data for the dominant leg of the able-bodied group. Significant differences due to speed (“^*^”) are indicated. The CoGx_mean_ and CoGy_mean_ (trajectory method) were examined for the clinical patient and it was found that while the x- and y- coordinates were similar (Table 7), the clinical participant showed more variability (Figure 6).

**Table 6:** CoG_x_ and CoG_y_ for the able-bodied group.

**Speed (°/s)**	**CoG_**x**_**	**CoG_**y**_**	**CoGx_**mean**_**	**CoGy_**mean**_**	**CoGx_**mean**_ var**	**CoGy_**mean**_ var**	**CoGx_**mean**_ range**	**CoGy_**mean**_ range**
60	6.73 ± 0.30	3.03 ± 0.09	6.67 ± 0.4	3.02 ± 0.02	0.070 ± 0.05	0.008 ± 0.01	1.1 ± 0.4	0.34 ± 0.2
90	6.72 ± 0.25	3.04 ± 0.08	6.76 ± 0.5	3.04 ± 0.04	0.18 ± 0.3	0.02 ± 0.02	1.3 ± 0.9	0.40 ± 0.3
120	6.75 ± 0.36	3.04 ± 0.08	6.75 ± 0.5	3.03 ± 0.03	0.07 ± 0.08	0.01 ± 0.01	0.95 ± 0.4	0.40 ± 0.2

**Table 7:** CoG_x_ and CoG_y_ for the clinical participant.

**Side**	**Speed (°/s)**	**CoG_**x**_ (mm)**	**CoG_**y**_ (mm)**	**CoGx_**mean**_**	**CoGy_**mean**_**	**CoGx_**mean**_ var**	**CoGy_**mean**_ var**	**CoGx_**mean**_ range**	**CoGy_**mean**_ range**
Affected (right)	60	6.7409	3.1035	6.6035	3.1229	0.0231	0.0012	0.6449	0.1636
	90	6.2881	3.1862	6.3071	3.1963	0.1934	0.0066	1.4416	0.2504
	120	6.0123	3.1852	6.2780	3.1783	0.2027	0.0057	1.5787	0.2703
Intact (left)	60	6.4424	3.0588	6.2667	3.1113	0.0572	0.0031	1.0368	0.1955
	90	6.8214	3.3013	6.6226	3.1073	0.2483	0.0063	1.6240	0.2572
	120	6.0192	3.1877	6.0910	3.0652	0.1299	0.0109	1.4699	0.3224

**Figure 7:**
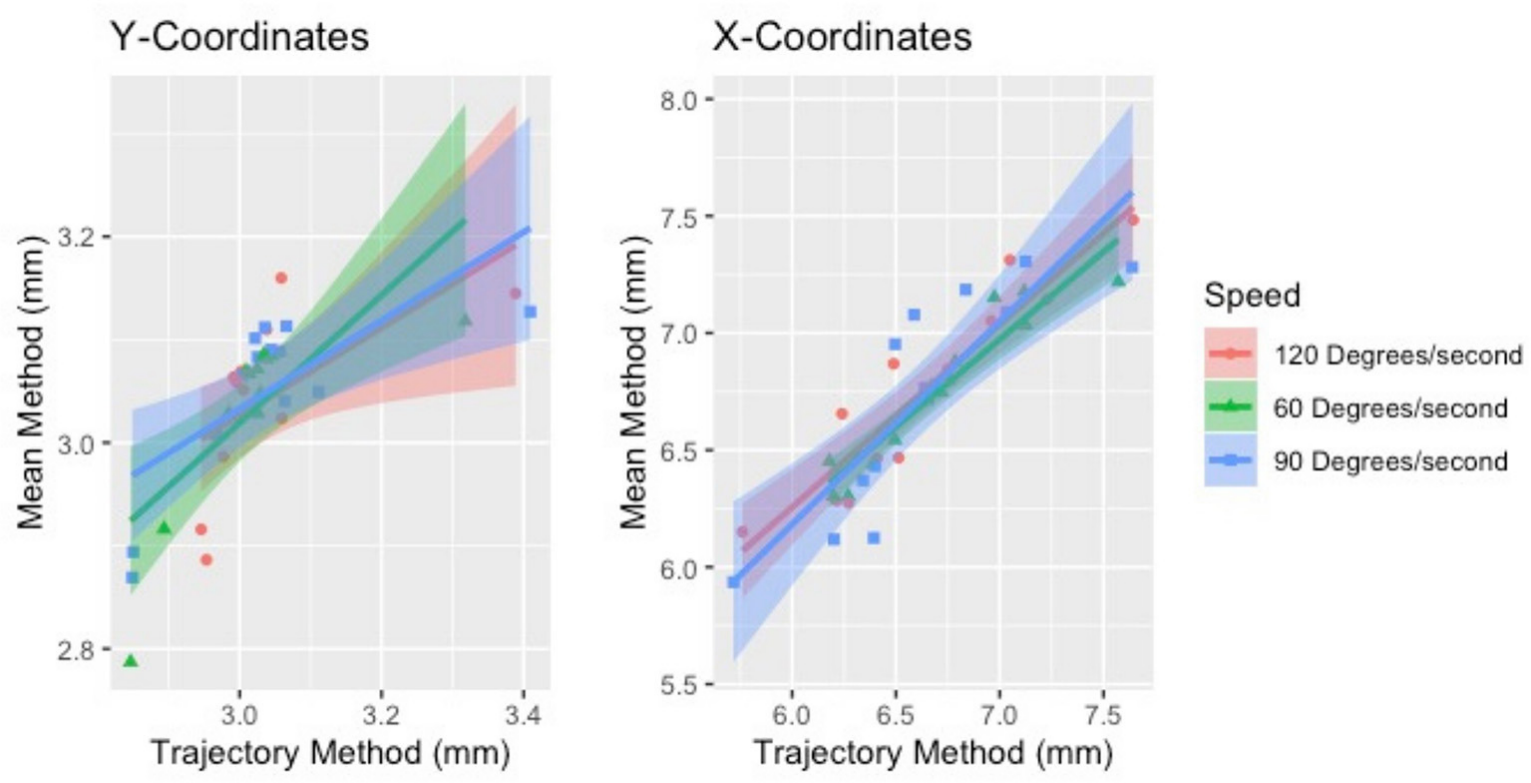
Correlation of the two methods of estimating CoG (mean and trajectory). The two methods were positively correlated across speeds for both sides.

The two methods of calculating CoG, mean and trajectory, were also compared against each other across all speeds using a Pearson product-moment correlation coefficient. A moderate to strong positive correlation was shown across speeds. The correlations for each coordinated (x and y direction) across speed are provided in Table 8. Correlation graphs are provided in Figure 7.

**Table 8:** Correlation between the two methods of CoG.

**Coordinate comparison**	**Side**	**Speed**	***r* ^**2**^**
**CoG**_**x**_ **M1 vs. M2**	*R*	60	0.95^**^
		90	0.86^**^
		120	0.93^**^
**CoG**_**y**_ **M1 vs. M2**	*R*	60	0.75^**^
		90	0.72^**^
		120	0.60^*^

* *Correlation (r^2^) is significant at the 0.05 level*.

** *Correlation (r^2^) is significant at the 0.01 level*.

## Discussion

HDsEMG can be used to develop spatial activity maps and features of these maps can be used to examine muscle function. In this study we used five features of the spatial maps to examine differences in intact and ampuated limb of a prosthesis user as well as in a control group. Slow, moderate and fast speed isokinetic knee extensions were completed while HDsEMG signals were collected from the rectus femoris muscle. The spatial features were compared between intact and ampuated limb after transtibial amputation. Features were also compared between a clinical participant and age-matched control participant.

For the clinical participant, it appeared that entropy decreased with increasing speed for the intact side. In addition, the CoV increased with speed on the intact side. The lower entropy and increased CoV suggest increased heterogeneity in the spatial HDsEMG potential distribution suggesting differences in motor unit strategies between the ampuated and intact limb. While caution must be taken with any conclusion with only one clinical participant, this suggests the need to further examine spatial parameters between intact and ampuated limb. There has been little research characterizing the EMG signal generated by the ampuated muscle (26). Furthermore, limited studies have used HDsEMG technology to understand differences between amputated and intact limb muscle function. Wentink et al. (36) compared muscle activity patterns from traditional bipolar surface EMG of those with transfemoral amputation to control participants during walking to better understand adaptions to gait patterns. Fylstra et al. (26) used HDsEMG and decomposition to show that during gait the HDsEMG signals presented modifiable signal features in amputated muscle compared to intact muscle and should be considered when developing myoelectric control schemes. Their work also highlights the need to investigate differences in spatial patterns of activation in both amputated and intact limb muscles in order to characterize muscle properties. Both of these previous studies examined gait with a focus on prosthesis control. Our present study focused on the performance of the lower limbs during isokinetic contractions to improve rehabilitation protocols for individuals with amputations. Furthermore, recent developments in HDsEMG include non-invasive decomposition techniques which can automatically extract motor unit discharges (37). In this work the contractions were rather short and future studies should examine longer muscle contractions to allow for muscle fiber decomposition to better understand motor function of amputated lower limb.

Table 5 presented the differences in torque and EMG data between the prosthesis user and age-matched control participant. Unsurprisingly, the prosthesis user had lower torque in his affected side than the non-dominant leg of the control participant. Surprisingly the prosthesis user had a higher peak torque on their sound side compared to the control participant. This was not expected as it has been previously found that individuals with transtibial amputation exhibit a decrease in strength on the sound side compared to a control group (4). In this study, the clinical participant was a healthy and active young male who suffered a traumatic amputation. His overall strength capability may simply have been greater than the age-matched control participant. However, they did not examine EMG in their work. For both the clinical and control participants, as speed increased, generally torque decreased. The age-matched control participant did show little change due to speed on their dominant leg.

Mean relative RMS was greater on the intact limb side compared to the affected side for the prosthesis user. In addition, the mean relative RMS was generally greater for the clinical participant compared to the age-matched control as were intensity and differential intensity indicating greater muscle activation. The median frequency appeared to be higher in the affected limb (71.3–80.2 Hz) compared to the intact limb (63.5–82.4 Hz) for the clinical participant. In addition, the clinical participant demonstrated lower median frequency during the slow and moderate contractions and higher median frequency during the fast contraction compared to the age-matched control participant. Median frequency is dependent on muscle geometry (38) which may partially explain these differences.

The spatial maps also demonstrated differences in activtation between the prosthesis user and age-matched control participant (Figures 2–5). Figures 4, 5 illustrated that the clinical participant had greater levels of muscle activity as demonstrated in the spatial maps of both the affected and sound side.

Surprisingly, there are few studies that have used HDsEMG to examine muscle activation patterns in those with amputated lower limbs and, in particular, both the intact and ampuated limb (24, 25). Although there has been promising work using EMG from the ampuated limb for myoelectric control, few studies have used this technology to characterize muscle activity (26). Of those studies that have used HDsEMG for lower limb examination, most have monitored muscle activity during gait (24) or isometric contractions (26). To our knowledge, there are no studies that have investigated ampuated muscle activity using HDsEMG during other dynamic activities such as isokinetic dynamometry. Isokinetic dynamometry is used routinely for rehabilitation, strength training and testing and is particularly well-suited for clinical populations.

Unfortunately, in this study, the HDsEMG was limited to one muscle of the quadriceps, the rectus femoris. The quadriceps muscle is comprised of three vastus muscles and the rectus femoris. The RF is a biarticular muscle that crosses the hip and knee joints while the vasti are one-joint muscles. While it has been shown that vasti and rectus femoris are all active during maximum isometric knee extension (39), it is possible that the HDsEMG parameters are different across the muscles. Furthermore, the torque that is collected from the dynamometer is a net torque and the EMG collected was limited to one muscle which may have affected the results. Future studies should examine all muscles of the quadriceps including the vasti. In addition, estimation of antagonist muscle may also provide further details of differences in intact and ampuated limb functioning.

In this work we also presented an additional feature of the centroid using a trajectory-based analysis using the entire contraction. The CoG data did show that the clinical participant had greater variability compared to the age-matched control participant (Figure 6). Currently, researchers use different methods to estimate the coordinates of the CoG and we examined a commonly used point method and introduced a new method of using the trajectory of the CoG throughout the contraction. The suggestion was that the use of the entire contraction rather than one single point at the peak intensity may influence the CoG.

Our results showed that the new method was highly correlated with the traditional CoG estimation method and, furthermore, speed had little effect. Previously it has been shown that regional changes can occur in muscle activation at different intensities (30) and it was expected that this may be reflected in the CoG. However, the CoG did not significantly change in the control group due to speed, regardless of estimation method. For the clinical participant there did not appear to be observable differences between the affected and intact limb. While this suggests that either method can be used, both methods used the same size window (250 ms) for data analysis. Previous research has used different window sizes for HDsEMG feature analysis depending on the movement studied (23, 27, 40). In addition, the nature of isokinetic contractions requires individuals to “push as hard as they can” throughout the contraction and therefore this may have limited the effects on the CoG variation.

Our results show that the HDsEMG can be used successfully with prosthesis users during isokinetic movements across a range of speeds. While this study was limited to one clinical participant, the differences noted would support the physiological differences between intact and ampuated limb. Furthermore, understanding these differences is critical to improve training protocols for those with amputation.

## Conclusion

This study showed that HDsEMG can be used to non-invasively examine the spatial distribution and neuromuscular physiology of those with amputation. The results from this exploratory study showed that spatial parameters can be used to provide insight regarding muscle function, particularly those representative of muscle heterogeneity. While limited in sample size, this preliminary data suggests that further investigation is warranted using HDsEMG features for this population, in particular with respect to aging muscle.

We examined two methods of estimating the CoG of the HDsEMG map and had equivocal results. Both the point and the trajectory methods yielded similar results and were correlated with one another. However, there remains a lack of consensus regarding the method to estimate this particular feature of the HDsEMG map. Future studies should further examine the window length as well as different algorithms to monitor this feature to move toward a more standardized methodology.

More comparative studies with a larger number of participants are needed to better understand the effect of transtibial amputation on the musculature of the lower limb. It is imperative to measure muscle activity from not only the affected side but also the sound limb in order to understand and gain insight into muscle compensations that may occur in this population. Furthermore, better muscle activation estimates can help clinicians develop appropriate training protocols to ensure improved neuromuscular function. The clinical participant in this study was young and healthy and had lived with an amputation for 7.5 years prior to taking part in the study. Further studies of individuals with congenital amputation as well as older adults dealing with the added burden of aging will help to better understand the effects of transtibial amputation on daily function.

## Data Availability Statement

The raw, unidentifiable data supporting the conclusions of this article will be made available by the authors, without undue reservation.

## Ethics Statement

The studies involving human participants were reviewed and approved by University of New Brunswick Research Ethics Board. The patients/participants provided their written informed consent to participate in this study.

## Author Contributions

UK, AP, and JT contributed to the conception and design of the study. AP developed the analysis software. UK, AP, and JT organized the data. JT performed the statistical analysis. UK wrote the first draft of the manuscript. AP and JT wrote sections of the manuscript. All authors contributed to manuscript revision, read, and approved the submitted version.

## Conflict of Interest

The authors declare that the research was conducted in the absence of any commercial or financial relationships that could be construed as a potential conflict of interest. The handling editor declared a past co-authorship with one of the author AP.

## Publisher's Note

All claims expressed in this article are solely those of the authors and do not necessarily represent those of their affiliated organizations, or those of the publisher, the editors and the reviewers. Any product that may be evaluated in this article, or claim that may be made by its manufacturer, is not guaranteed or endorsed by the publisher.
